# Lettuce (*Lactuca sativa* L.) Cultures and the Bioactivity of Their Root Microflora Are Affected by Amended Soil

**DOI:** 10.3390/plants13131872

**Published:** 2024-07-06

**Authors:** Konstantina Mitsigiorgi, Georgia C. Ntroumpogianni, Efstathios A. Katsifas, Dimitris G. Hatzinikolaou, Konstantinos Chassapis, Elisavet Skampa, Aikaterina L. Stefi, Nikolaos S. Christodoulakis

**Affiliations:** 1 Section of Botany, Department of Biology, National and Kapodistrian University of Athens, Panepistimiopolis, 15784 Athens, Greece; mitsig@biol.uoa.gr (K.M.); georgiantr@biol.uoa.gr (G.C.N.); skatsi@biol.uoa.gr (E.A.K.); dhatzini@biol.uoa.gr (D.G.H.);; 2 Inorganic Chemistry Laboratory, Department of Chemistry, National and Kapodistrian University of Athens, Panepistimiopolis, 15771 Athens, Greece; chassapis@chem.uoa.gr; 3 Section of Historical Geology–Paleontology, Department of Geology and Geoenvironment, National and Kapodistrian University of Athens, Panepistimiopolis, 15771 Athens, Greece; elskampa@geol.uoa.gr

**Keywords:** *Lactuca sativa*, soil improvers, organic fertilization, rhizosphere, antimicrobial activity

## Abstract

This study aimed to highlight the positive effects of various recycled organic substrates on lettuce plants (*Lactuca sativa* L.) and to promote sustainable waste management practices, contributing to the concept of a circular economy. Over a two-month period, the growth potential and rhizosphere microflora of lettuce plants grown in soil amended with different recycled substrates were investigated. All data were compared, and the effects of the culture substrates were evaluated. All groups containing soil improvers offered a significant increase in the number of leaves per plant and, in two cases, an increase in dry biomass as well as an increase in the concentration of all leaf pigments. Both MDA and H_2_O_2_ concentrations were the lowest in two groups containing soil improvers (VG 5% and PLUS 10%). At the end of the culture period, isolation and culture of bacteria from the plant rhizosphere were performed. Different bacterial strains were isolated and tested for the production of antimicrobial agents against six microbial indicators (*B. subtilis*, *E. coli*, *S. aureus*, *S. cerevisiae*, *C. albicans*, and *P. aeruginosa*). The greater percentage of the isolated strains showed an ability to inhibit the growth of the *B. subtilis* index. Most of the strains with antimicrobial activity were isolated from the soil samples of the plain soil group and the soil amended with the commercial fertilizer. Three of the isolated strains originating from the Ginagro 5% group are multiproducers as they inhibit the growth of three microbial indicators or more.

## 1. Introduction

Chemical fertilizers have been used in the past decades as the main method of crop fertilization. However, excessive use can decrease the soil quality by reducing the soil pH and accelerating the decomposition of organic matter [1], as well as nutrient leaching into the aquifer [2]. In addition, it may affect the soil microbial community composition and their function [1]. Apart from the environmental impact, the effort to limit the use of inorganic fertilizers also derives from the rising prices of fertilizers [3]. Organic fertilizers are a promising alternative that also maintains a high interest in the scientific community, as multiple aspects regarding the effects they have are not yet fully understood. 

The product group “soil improvers” has been defined as “materials to be added to the soil in situ in order to maintain or improve its physical, chemical, and/or biological properties or activity” by the 2022/1244/EU Commission’s Decision [4] of the 13 July 2022 for the award of the Community eco-label to soil improvers and growing media. The composition of each soil improver depends on its origin and method of preparation. Concerning the soil improvers originating from processed plant residues, they may contain factors known for their bio-stimulant or photo-regulatory effect (i.e., humic acids) [5]. Soil improvers and their components are found to accelerate the growth rate and seedling survival [6] and promote the development of lateral roots [5]. There is also evidence that they can enhance a plant’s tolerance to salinity stress [7] and modify metabolic pathways [8]. Regarding the effects of soil improvers on soil properties, it is justified that the application of soil improver ameliorates the soil’s structure, aeration, and water holding capacity [9], as well as the organic matter content [6], while supplying nutrients necessary for plant growth [10]. It also promotes soil microbial growth and activity [11].

Another major advantage of soil improvers, in contrast to inorganic fertilizers, is their beneficial role to sustainable agriculture. Soil improvers of assured quality fulfill the requirements of a sustainable cycling of organic materials in an optimal way, regarding ecologic and economic aspects [10]. Soil improvers deriving from green waste and biodegradable municipal waste contribute to the reduction of greenhouse gas emissions by reducing the emissions from landfills and the construction of inorganic fertilizers [12]. Additionally, the use of soil improvers might reduce the need for use of pesticides [12] and can provide a solution to the loss of land caused by salinization and drought [3].

The rhizosphere is the area of close interaction of the plant with soil microorganisms. It has been documented that this area is directly affected by the secretions of the plants’ roots [13]. Bacteria isolated from the rhizosphere secrete a rich variety of secondary metabolites and have been a subject of interest for many years. The production of secondary metabolites, several of which have antimicrobial activity, is the result of interspecific interactions and competition with biotic factors for nutrients and stressors [14]. Studies revealed that the structure of the soil bacterial community is often significantly affected by the application of different fertilizers and soil improvers. In addition, it has been demonstrated that individual fertilizers and soil improvers remarkably affect the antimicrobial activity of these bacteria [15].

Lettuce (*Lactuca sativa* L.) is a plant species cultivated worldwide, with great economic importance. Its significance for human nutrition triggered multiple studies, especially concerning the effect of soil improvers and fertilizers as well as the optimization of its growth rate [16].

The current investigation was launched using soil improvers deriving from municipal waste of tree pruning residues and organic kitchen waste. It aspires to showcase that waste materials can be utilized as a valuable resource for agriculture, demonstrated on a widely cultivated plant species. This approach not only minimizes the environmental footprint of agricultural practices but also encourages municipalities and communities to adopt more sustainable waste management strategies. Consequently, the use of recycled organic substrates fosters a closed-loop system where waste is repurposed, supporting both ecological balance and economic resilience.

## 2. Results

### 2.1. Productivity

**Figure 1:**
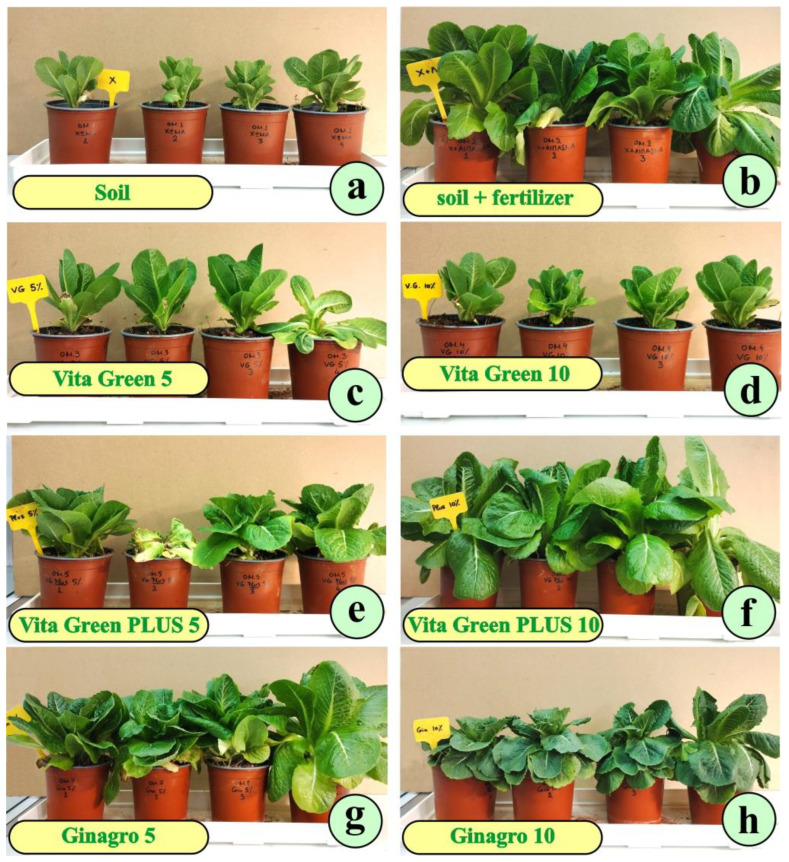
Plants from the eight culture groups, after the end of the culture period. The labels, lower left in each picture, indicate the composition of the culture substrate. (**a**) lettuce plants cultured in plain soil; (**b**) plants in soil +fertilizer; (**c**) plants cultured in soil amended with Vita Green 5%; (**d**) plants cultured in soil amended with Vita Green 10%; (**e**) the culture substrate is soil amended with Vita Green + 5%; (**f**) the culture substrate is soil amended with Vita Green + 10%; (**g**) lettuce plants cultured in soil amended with Ginagro 5% and (**h**) plants in soil with Ginagro 10%.

At first glance, we realized that the growth potential of the plants varied significantly according to the culture substrate. The lettuce plants cultivated in plain soil appeared weak (Figure 1a). When the soil was boosted with commercial fertilizer, the development of the plants was boosted as well (Figure 1b). Vita Green 5% (VG 5%) (Figure 1c) and Vita Green 10% (VG 10%) (Figure 1d) substrates did not seem to favor lettuce growth. Vita Green Plus 5% (PLUS 5%) (Figure 1e) did not give impressive results, while Vita Green Plus 10% (PLUS 10%) replaced the commercial fertilizer with a similar outcome (Figure 1f). Ginagro 5% (GIN 5%) (Figure 1g) strongly promoted plant growth, while Ginagro 10% (GIN 10%) (Figure 1h) seemed to weakly support the plants.

**Figure 2:**
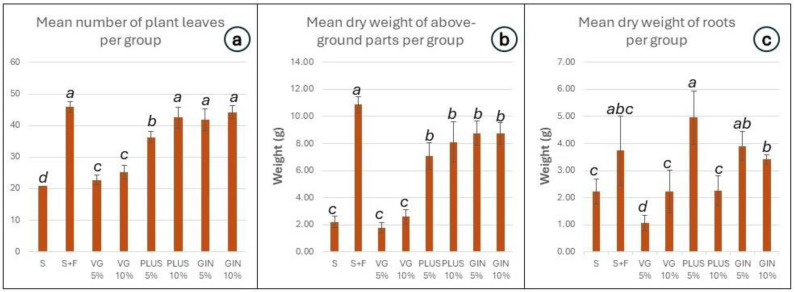
(**a**) The number of plant leaves per group. (**b**) The dry weight (biomass) for the above-ground parts per group. (**c**) The dry weight (biomass) of the roots per group after the end of the experiment. Error bars represent the standard error of the mean; the results are grouped using the compact letter display (CLD) methodology; *p* values ≤ 0.05 were characterized as statistically significant.

Concerning the productivity of the various culture treatments, we observed and recorded the following three parameters: (a) the number of the leaves; (b) the leaf biomass; and (c) the root biomass. All groups containing soil improvers demonstrated a statistically significant increase in the number of leaves per plant in comparison to plain soil (S). Three groups (PLUS 10%, GIN 5%, and GIN 10%) had a leaf number per plant that is close to that of the fertilized soil (S + F) (Figure 2a). In terms of leaf dry biomass, all PLUS and GIN groups had greater biomass than that of the plants grown in plain soil (Figure 2b), while the plants grown in fertilized soil exhibited a statistically significant increase in biomass compared to all other groups. The dry biomass of the roots increased in the groups of PLUS 5%, GIN 5%, GIN 10%, and S + F, a characteristic that is desirable in water deficiency conditions. The groups of PLUS 10% and VG 10% seemed to have no statistical difference compared to the S group, while plants grown in the VG 5% demonstrated a smaller root dry biomass compared to all other groups (Figure 2c).

### 2.2. Leaf Anatomy and Microscopy

**Figure 3:**
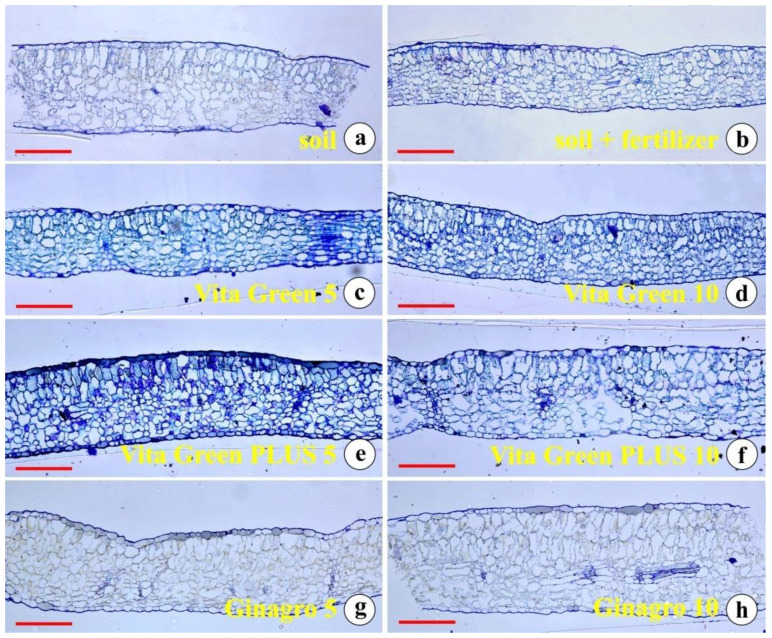
Cross-sections of epoxy embedded leaves from plants growing in (**a**) plain soil; (**b**) soil with conventional fertilizer; (**c**) Vita Green 5%; (**d**) Vita Green 10%; (**e**) Vita Green PLUS 5%; (**f**) Vita Green PLUS 10%; (**g**) Ginagro 5%; and (**h**) Ginagro 10%. Scale bars in all micrographs are 100 μm.

Concerning the anatomy of the mature leaves, we could easily observe the differences between the plants cultured on various substrates. The mesophyll tissue construction as well as the thickness of the epidermal cells and their cell walls varied between the different groups. Palisade and spongy parenchyma were rather loose and not well defined in the leaves of the plants cultured in plain soil (Figure 3a). Plants cultured in soil amended with commercially available conventional fertilizer were more compact, particularly in their spongy parenchyma (Figure 3b). The leaves of the plants grown in VG 5% were thin, with rather loose mesoplyll and thin cell walls in general (Figure 3c).

Leaves from the plants grown in VG 10% (Figure 3d) appeared rather similar to the leaves of the previous group. PLUS 5% (Figure 3e) and PLUS 10% (Figure 3f), developed thick leaves with a compact mesophyll. Only a few intercellular spaces appeared while their epidermal cells were thicker, accumulated metabolites, and possessed well-defined cell walls. The leaves of the plants cultured in GIN 5% (Figure 3g) and GIN 10% (Figure 3h) appeared to be superior in thickness with thinner epidermal cell walls and rather loose histological structure.

**Figure 4:**
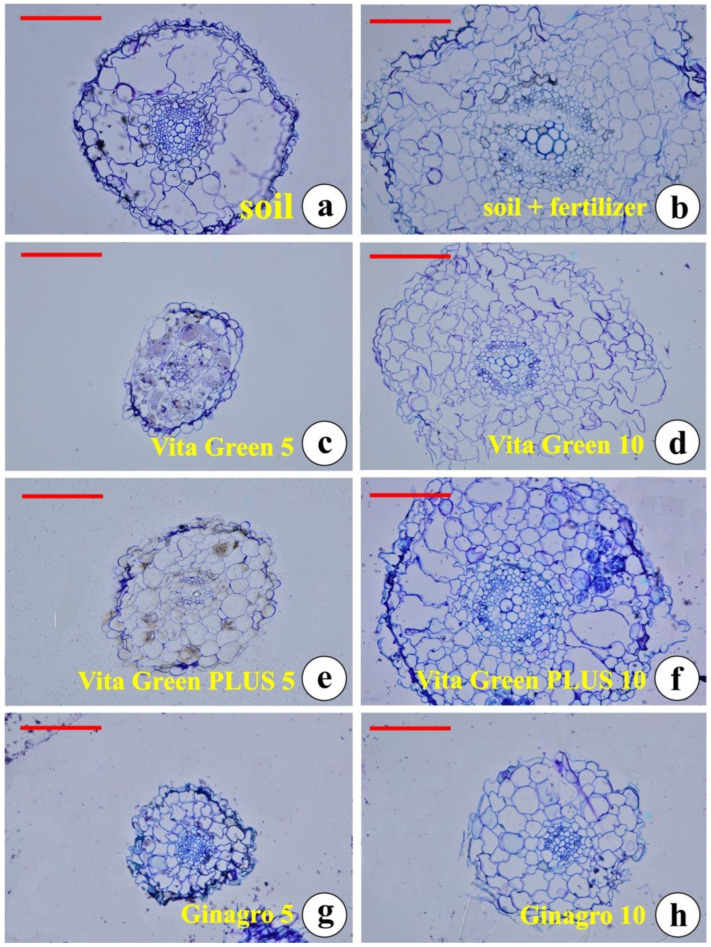
Cross-sections of epoxy embedded roots from plants growing in (**a**) plain soil; (**b**) soil with conventional fertilizer; (**c**) Vita Green 5%; (**d**) Vita Green 10%; (**e**) Vita Green PLUS 5%; (**f**) Vita Green PLUS 10%; (**g**) Ginagro 5%; and (**h**) Ginagro 10%. Scale bars in all micrographs are 100 μm.

Observing the cross-sections of epoxy embedded roots from plants growing in various substrates (Figure 4), we can assume that, although the development of the root—according to the distance from the root tip—may vary, the structure of the conductive tissue and the arrangement of the endodermis are similar. We observed two (Figure 4b,f) and three (Figure 4a,d) of xylem elements, while in younger roots the xylem has not been arranged yet (Figure 4c,e,g,h).

**Figure 5:**
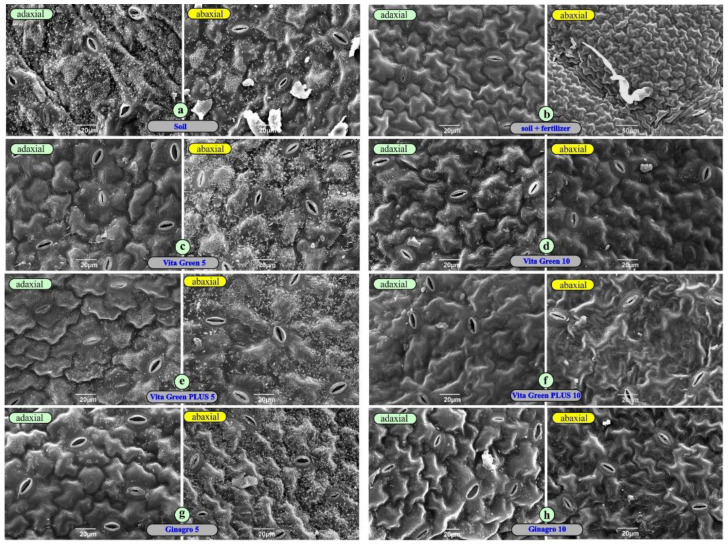
Both surfaces of a leaf from a plant growing in (**a**) plain soil; (**b**) soil with conventional fertilizer; (**c**) Vita Green 5%; (**d**) Vita Green 10%; (**e**) Vita Green PLUS 5%; (**f**) Vita Green PLUS 10%; (**g**) Ginagro 5%; and (**h**) Ginagro 10%. Some trichomes and stomata can be observed. The stomatal frequency is given in the text as number of stomata/mm^2^.

Scanning electron microscopic observations on the surface topography of the various leaf types (Figure 5) revealed a rather uniform structure accommodation of the epidermal cells. The number of stomata on the abaxial surface always appeared superior than that of the lower surface. Concerning the stomatal frequency we counted the number of stomata/mm^2^ for plain soil (Figure 5a) 222 ± 11 for the upper and 245 ± 9 for the lower epidermis; for S + F (Figure 5b) 99 ± 9 for the upper and 107 ± 8 for the lower epidermis; for VG 5% (Figure 5c) 80 ± 11 for the upper and 92 ± 7 for the lower epidermis; for VG 10% (Figure 5d) 172 ± 11 for the upper and 202 ± 6 for the lower epidermis; for PLUS 5% (Figure 5e) 42 ± 8 for the upper and 65 ± 11 for the lower epidermis; for PLUS 10% (Figure 5f) 182 ± 9 for the upper and 204 ± 12 for the lower epidermis; for GIN 5% (Figure 5g) 153 ± 8 for the upper and 172 ± 12 for the lower epidermis; and, finally, for GIN 10% (Figure 5h) 102 ± 8 for the upper and 127 ± 11 for the lower epidermis. Standard deviation of the stomatal frequency was calculated using one-way ANOVA. Besides the expected difference of the stomatal frequency between the adaxial and the abaxial epidermis, no other valuable data were revealed from the SEM observations.

### 2.3. Photosynthetic Pigments

**Figure 6:**
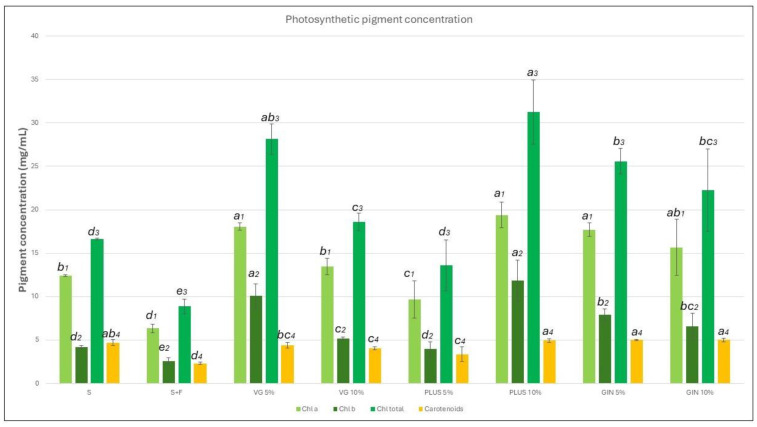
Bar graphs depicting the concentration of chlorophyll a (●), chlorophyll b (●), total chlorophylls (●), and carotenoids (●). Error bars represent the standard error of the mean; the results are grouped using the compact letter display (CLD) methodology; *p* values ≤ 0.05 were characterized as statistically significant.

Spectrophotometric measurement of the leaf pigments demonstrated an increased pigment content in all soil amended groups. The concentration of chlorophylls a and b as well as carotenoids was calculated, as seen in Figure 6. Plants grown in fertilized soil seemed to have the least amount of chlorophyll a, chlorophyll b, and carotenoids across all groups. Plants grown in plain soil contained fewer total chlorophylls than all soil improver groups. However, the carotenoid concentration was higher in relation to most other pigment groups, surpassing the concentration of chlorophyll b. VG 5% and VG 10% groups had a higher chlorophyll concentration, yet less carotenoids when compared to the plain soil group. The PLUS 5% group was counted to have a concentration similar to the S group and slightly lower than the VG 10% group. A significant increase in the concentration of all pigments was observed in the PLUS 10% group, which had the highest concentration of all pigments in comparison to all other groups. Both GIN groups also demonstrated a high pigment concentration both in chlorophyll and carotenoid contents (Figure 6).

### 2.4. Oxidative Stress

**Figure 7:**
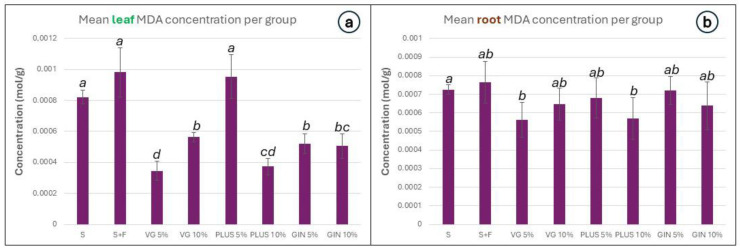
Bar graphs depicting measured MDA concentration in (**a**) the leaves and (**b**) the root of *L. sativa*. Error bars represent the standard error of the mean. The results are grouped using the compact letter display (CLD) methodology; *p* values ≤ 0.05 were characterized as statistically significant.

**Figure 8:**
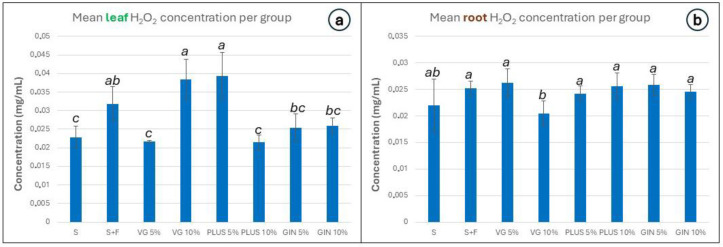
Bar graphs depicting measured H_2_O_2_ concentration in (**a**) the leaves and (**b**) the root of *L. sativa*. Error bars represent the standard error of the mean. The results are grouped using the compact letter display (CLD) methodology; *p* values ≤ 0.05 were characterized as statistically significant.

Oxidative stress was estimated by the malondialdehyde (MDA) method as well as by measuring the hydrogen peroxide (H_2_O_2_) concentration. The difference in concentration between the groups was greater in the leaves for both stress indicators (Figure 7a and Figure 8a). Concerning MDA, its concentration was smaller in all soil improver groups except for PLUS 5%, in comparison to S and S + F in the leaves. The lowest MDA concentration was observed in the VG 5% and PLUS 10% groups (Figure 7a). The concentration was slightly lower in the roots of all soil improver groups, with a statistically significant smaller concentration in the groups VG 5% and PLUS 10% in relation to plain soil (Figure 7b). 

The concentration of H_2_O_2_ was higher in the leaves of the S + F, VG 10%, and PLUS 5% groups. The lowest concentration was observed again in the groups VG 5% and PLUS 10%. The roots of all groups seemed to have a similar amount of H_2_O_2,_ with the VG 10% group presenting a slight decrease (Figure 8).

### 2.5. Antimicrobial Activity

**Figure 9:**
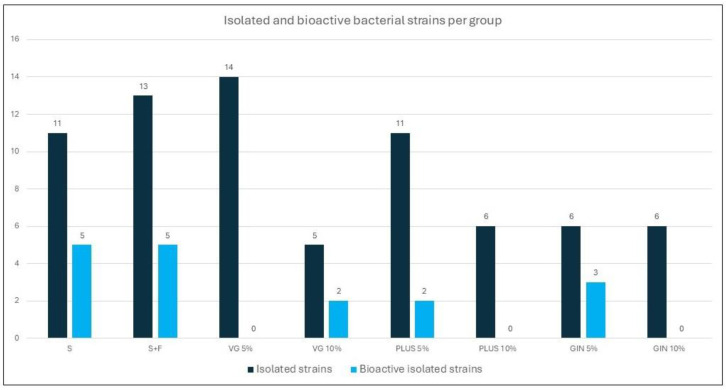
Total number of isolated strains from each soil sample (●) and the number of strains with antimicrobial activity (●).

Seventeen out of seventy-two morphologically distinct bacterial strains isolated from the eight different soil samples of the experiment presented antimicrobial activity against at least one of the six indicator strains (Figure 9). As it is demonstrated, none of the strains isolated from the groups VG 5%, PLUS 10%, and GIN 10% had the ability to inhibit the growth of any microbial indicator.

Thirteen out of the seventeen bioactive strains (76%) inhibited the growth of *B. subtilis*, six (35%) of *C. albicans*, three (18%) of *S. aureus*, three (18%) of *S. cerevisiae*, one (6%) of *P. aeruginosa,* and one (6%) of *E. coli.* The ATHUBA 2670, ATHUBA 2671, and ATHUBA 2674 strains, which were isolated from the GIN 5% group, were characterized as multi-producers as they inhibited the growth of three microbial indicators or more. In particular, the ATHUBA 2674 strain inhibited the growth of all microbial indicators (Table 1).

## 3. Discussion

Chemical fertilizers and pesticides are widely used in agriculture; these agrochemicals impose an extra major pressure or/and a threat to our environment, resulting in soil quality degradation; pH becomes more acidic; a low concentration of organic matter makes no secure to food quality/safety [17]. Furthermore, there is also an impact on the economy, as production costs are getting higher [18,19,20]. On the other hand, waste management emerges as a major concern that people must cope with, as its proper use may contribute to the implementation of municipal policies towards waste recovery and reuse [21]. Creation of sustainable agricultural systems producing food of good quality without imposing threats to environmental resources is the aim of new agricultural practices [22]. Under this scope, the use of soil improvers from municipal waste has emerged as the new practice [23]. *Lactuca sativa*, a vegetable combining low price and delicious taste as well as high nutritional value, is widely consumed worldwide [24]. 

After the detailed investigation on the effect of soil amendment with certain, eco-friendly, locally produced materials, we may assume that a significant promotion of plant development can be achieved with inconsiderable expenses and a great benefit for the environment. 

Our data indicate that, among the improvers used, VitaGreen5+, VitaGreen10+, Ginagro5, and Ginagro10 significantly promote the development of lettuce plants. The structure of the leaf and the epidermis, the accommodation of the leaf tissues, and the structure of the root are not affected, in general. The rest of the improvers do not seem to augment the crop but can easily be used as common fertilizers since their cost is negligible for the farmer. It seems that, through this investigation, a new source of recycled materials can be proposed for use in environmentally friendly farming. Furthermore, in order to explain if the new environment with soil improvers introduces stressful conditions, MDA and H_2_O_2_ measurements were performed, in particular the following:

Vita Green. Concerning the use of soil amended with VG 5% and VG 10%, we observe that the lettuce plants cultured on these substrates have the same number of leaves and the same biomass of the root and the shoot as the plants cultured on plain soil. The MDA values for the above two groups of plants were counted at lower levels than those in the plain soil and soil + fertilizer groups. H_2_O_2_ remains at low levels in the leaves of the VG 5% plants, but it rises in the VG 10% group as compared to the plain soil and soil + fertilizer groups. The same is true for the roots. The photosynthetic pigments, particularly the chlorophylls, are measured at higher levels in the leaves of the VG groups when compared to those of the S and S + F groups. The VG 10% group, compared to the VG 5%, exhibits increased leaf number, increased biomass, and ROS-H_2_O_2_ in their leaves, yet their photosynthetic pigments are reduced.

Generally, VG 5% and VG 10% groups produce similar biomass with the plain soil group. However, we observed an increase in pigment content as well as a decrease in MDA and H_2_O_2_ concentration. This indicates that while this soil improver is beneficial for plant growth, perhaps either the concentration of the improver is not high enough to significantly alter the plant biomass or it is not an ideal substrate for lettuce growth.

Vita Green PLUS. The plants cultured on the Vita Green PLUS 5% amended soil develop more leaves and produce more biomass than the plain soil and the soil + fertilizer groups. The pigment concentration equals that in the leaves of the plants cultured on the plain soil; similar value of MDA but higher value of H_2_O_2_ in the leaves compared to the plain soil and soil + fertilizer groups. The plants cultured on the Vita Green PLUS 10% amended soil develop more leaves and total biomass compared to the plain soil and the soil + fertilizer groups. The pigment concentration is higher, while the MDA and H_2_O_2_ of the leaves are lower compared to the plain soil and soil + fertilizer groups. Vita Green PLUS soil improver significantly promotes the production of biomass and the development of thicker, more compact leaves. This soil improver derives from plant trimming and food waste and has a higher content of total nitrogen and nutrient availability (Table 3, see Section 4). In the higher concentration (Vita Green PLUS 10%), apart from the increase in biomass, an increase in photosynthetic pigments can also be observed, along with a significant decrease in MDA and H_2_O_2_ concentration.

Ginagro. The plants cultured on the Ginagro amended soil develop more leaves and biomass than their counterparts cultured on the plain soil. Lower MDA values were recorded. H_2_O_2_ concentration is higher, while the photosynthetic pigments appear to be increased compared to the plain soil and soil + fertilizer groups. Fewer microbial strains were isolated from the soil amended with Ginagro 5%, yet three of them present bioactivity against three or more bioindicators. Ginagro 10% totally lacks bioactive microorganisms. 

Briefly, we could say that both Ginagro groups exhibit increased biomass, leaf thickness, and pigment content. Both MDA and H_2_O_2_ concentrations are lower than, but not as low as, the PLUS10 group.

Reactive oxygen species (ROS) are commonly attributed to stress in plants. They result in reduced energy metabolism or disrupted membrane stability, as well as to plant productivity [25,26]; many Mediterranean plants’ “habit” response to any stressor is ROS production [27,28,29]. In the current experiment, most soil improvers decrease the content of oxidative stress markers (Figure 7 and Figure 8) in comparison to the fertilized soil group. In particular, the groups PLUS 10%, GIN 5%, and GIN 10% exhibit the lowest marker concentration in the leaves while also producing the highest amount of biomass in the above-ground parts of the plant.

The difference in plant survival and growth amongst the different treatments might derive from the regulatory effect of substances like phytohormones that are present in the soil improvers, as well as the nutrient availability. The average content of plant hormones has been found to be higher in soil improver mixtures containing larger amounts of plant waste [30]. Plant biomass, on the other hand, depends heavily on nutrient availability, and the soil improver that promoted the accumulation of biomass the most is the one that also contains food waste (Vita Green Plus), which has a higher content of total nitrogen and nutrient availability (Table 3, see Section 4). According to the literature, an increase in total biomass of lettuce was observed when a dry powder of the algae *Chlorella vulgaris* was applied to soil with lettuce sprouts [31], while the cultivation using the commercial algae product Maxicrop^®^ (*Ascophyllum nodosum*, Maxicrop USA, Inc., Elk Grove Village, IL, USA) resulted also in improvement of total lettuce yield [32]. In addition, the increase in the number of leaves as well as in fresh leaf mass was the result of cultivation of lettuce seedlings in poultry manure and sawdust [22], while chicken manure led to the augmentation of lettuce yield [33]. Furthermore, lettuce cultivation in a green-tea waste substrate resulted in cultivars with increased numbers of leaves [34]. Enrichment of soil with organic soil improvers could also regulate the flow of heavy metals; specifically, the addition of vermiculite, a phyllosilicate mineral consisting of Si_2_O_5_, resulted in reduced heavy metal uptake of lettuce and an improvement of biomass and average quality of the product [35]. The same is true for another gemstone. Tourmaline, a cyclosilicate, when applied to soil with lettuce, decreased the uptake of cadmium and copper by the shoots [35] and increased the concentration of nutrients in the soil [36]. 

Microorganisms represent the essential living part in the soil. Bacteria, actinomycetes, fungi, algae, and microfauna regulate the quality of the soil [37], contributing to the immobilization and mineralization of any nutrients, regulating the equilibrium between stable and non-stable agricultural cultivation [38,39]. Furthermore, due to their role in nitrogen fixation and secretion of metabolites that inhibit pathogens, they are in close contact not only with the plants’ roots but also with their development in a “fine-tuning mode” [37]. Consequently, when the soil becomes enriched by improvers, fluctuations in the microbiota community are observed [40]. 

By comparing the microflora from Kalamata soil samples with fertilizer or soil improvers with the microflora from the sample of plain Kalamata soil, it appears that the enriched soil samples constitute an environment for the growth of more microorganisms with antimicrobial activity, specifically microorganisms that can inhibit the growth of either Gram-positive or Gram-negative bacteria or fungi or a combination or all of these. In a recent study, compost of diverse green wastes resulted in increased concentrations of microorganisms and their activity [41]. 

Soil pH is another factor that affects both biomass and yield of plants [42], because it affects biogeochemical processes in the soil in a bidirectional relationship. Solubility of organic compounds is controlled by soil pH, either by effect on microbial activity or on humic acid charge density [43,44]. Therefore, soil pH regulates the maintenance/change in microbial community; it is characteristic that microbial metabolic quotient (MMQ) is much higher in low-acid soils compared to neutral pH soils [45]. In our data, Kalamata soil with Ginagro has a higher pH than the rest soil samples (low alkaline; Table 4, see Section 4); these conditions could be characterized as stressing conditions for the microflora, and a shift in the bacterial metabolism will be required for survival [46]. This alkaline pH, resulting in the presence of the most multi-producer strains, may be the cause for the production of antimicrobial substances and also the absence of fungi and lichens on the soil surface in the Kalamata soil with Ginagro. 

Furthermore, bacteria with antimicrobial activity (almost 99% of them) could produce at least one bacteriocin [47]. Data presented here, examining the microflora and the antimicrobial activity of the bacterial strains, reveal that the majority of the strains with antimicrobial activity inhibit the growth of *B. subtilis* (Table 1). In plain soil (“Kalamata”), out of the total of eleven bacterial strains isolated, five exhibit antimicrobial activities against either *C. albicans* or *B. subtilis* indicator strains. A total of thirteen strains were isolated from the Kalamata soil with commercial fertilizer (Complesal^®^ Supra, Veterin, Industrial and Commercial S.A., Athens, Greece), of which five strains had antimicrobial activity, all against *B. subtilis.* A total of nineteen bacterial strains are isolated from Kalamata soil amended with Vita Green soil, but only two had antimicrobial activity. A total of 17 strains were isolated from the Kalamata soil with Vita Green Plus; only 2 had antimicrobial activity. Finally, a total of twelve strains are isolated from Kalamata soil with Ginagro, but only three of them have antimicrobial activity. Of these three strains, ATHUBA 2670 and ATHUBA 2671 inhibit the growth of *B. subtilis*, *C. albicans*, and *S. cerevisiae*, and ATHUBA 2674 inhibits the growth of all microbial indicators. 

In general, organic soil improvers, including manures, crop residues, and sewage sludge [48,49,50,51,52], affect the activity of microorganisms, while they offer monomers or/and polymers, such as carboxylic acids, carbohydrates, phenolics, amino acids, etc., to them [53]. Due to their immediate response to the new environment [54], it is really necessary to try to understand their response to the new environment and to correlate it with the developmental procedure of the plant. The latter will help farmers to better monitor the soil quality and, therefore, the growth of the plant. Organic soil improvers could change even the 69% of the microbiota community, enhancing their diversity [39]. As a result, this new diversity affects processes of nitrification, denitrification, and nitrogen fixation in the soil; a 15-year study by Li et al. [53] of soil enrichment by manure increased gradually the microbial population of the soil, an effect that has not been noted by using inorganic fertilizers. However, we must always bear in mind that the same soil improvers may affect microbiota in a different way in different soil types [40]. Furthermore, a simile of Emeritus Prof. Dr Eric Van Ranst, Ghent University, refers to “soil pH is like the temperature of a patient during a medical diagnosis”, implying that pH informs us immediately about the conditions and microbiota in soil. Thus, in our experiments and agricultural practices, we must always have a place in the equation for soil pH [44]. 

Finally, it seems well documented that soil improvers, among many other benefits, do have a lower cost for the crop, are friendly to the environment, and improve the structure of the soil. The current research is based on a practical example of municipal waste management, according to which the farmer obtains the soil improvers free of charge or at a very low cost while, at the same time, the municipality overcomes the problem of waste accumulation in a green, sustainable way.

Our experiments demonstrate the beneficial role of the soil improvers used not only in biomass production but also in relieving stress and supporting the development of beneficial microorganisms that can boost plant defense against pathogens. All these are positive steps on behalf of a “greener” environment and an “active” bioeconomy.

## 4. Materials and Methods

### 4.1. Soil and Compost

A loamy soil (Table 2) was collected from an agricultural area of Kalamata, Peloponnese, Greece (37.059456 N, 22.109166 E). It was transferred in sterile plastic bags to the laboratory, where it was air-dried. Then, the soil was passed through a 2 mm sieve and kept for further analysis. All data from soil analyses are included in Table 2.

Particle size distribution was determined by the hydrometer method [55], and cation exchange capacity after the extraction of cations was determined by atomic absorption spectrometry [56]. Organic carbon concentration was measured by bichromate oxidation according to Walkley–Black’s procedure [57]. Soil pH was measured in a 1:1 (*w*/*v*) soil/water sludge [58]. The available phosphorus concentration was determined using the Bray–Kurtz method [59]. The exchangeable cations Ca^2+^, Mg^2+^, K^+^, and Na^+^ were determined by the NH-acetate method [60], and total N was titri-metrically measured after the distillation of NH_3_ using the Kjeldahl digestion [61]. Spectrophotometry was used to quantify P after using the Bray–Kurtz method (Milton Roy; Spectronic 401, Ivyland, PA, USA), K^+^ and Na^+^ were quantified by flame emission spectroscopy (Corning, Flame Photometer 410, Corning, NY, USA), and Ca^2+^ and Mg^2+^ by atomic absorption spectrophotometry (Varian SpectrAA 300). The results were expressed in dry weight. Calcium carbonate equivalent was tested using a Bernard calcimeter [62], but the result of the reaction was negative. At all stages of sample preparation and analysis, stringent precautions were taken to minimize contamination through air, glassware, and reagents.

The soil improvers employed in this investigation originated from organic waste of the municipality of Vari–Voula–Vouliagmeni, SE of Attiki (Vita Green and Vita Green Plus), and cotton ginner plant waste from G&P Cotton (Ginagro) via biocatalytic processing. Their main properties, as declared on the package by the manufacturer, are displayed in Table 3.

### 4.2. Plant Material, Experimental Set-Up, and Culture Conditions

The plants were allowed to grow for a total of 60 days, after which the experiment was terminated and plant samples were collected. Seeds of *Lactuca sativa* var. Parris Island, purchased from Gemma (Gemma, GR), were imbibed at 24 °C for 24 h. Furthermore, they were left to germinate on moist (distilled water) filter paper in Petri dishes. The Petri dishes were placed in a P-Selecta incubator (Model No. 2000238, Barcelona, Spain), ventilated through a HAILEA ACO-9160 (Guangdong Hailea Group Co., Ltd., Chaozhou, Province, China), at an output of 4 L/min., at 25 °C with a light/dark cycle of 16 h/8 h (Philips CorePro LED bulb, 11.5 W = 75 W, at 2700 K, 105 mA) producing 2500 lx radiation (Photosynthetically Active Radiation = 60 μmol m^−2^ s^−1^). After 5 days, the radicle appeared (95% of the seeds were germinated). Then, the seedlings were sown in 8 groups, 10 pots for each group, with 1 seedling in each pot (10 plantlets/treatment). 

Each group was placed on a separate tray within a growth chamber at 25 °C. The tray was watered daily through an irrigation timer. The growth chamber was equipped with 4 LED panels (150 W; 3000 K) and 8 LED Grow Bulb-SMD 2835 (160 W; 640 nm and 460 nm). The photosynthetic photon flux density (PPFD) in the growth chamber was measured to be 800 µmol m^−2^ s^−1^ after a light/dark cycle of 16 h/8 h.

In each treatment, the soil was enriched with a different type of soil improver. The substrates for each group are presented in Table 4. Soil pH was measured at the termination of the experiment, as formerly described, in a 1:1 (*w*/*v*) soil/water sludge [58]. Soil pH is presented in the last column of Table 4.

Then, the pots were removed for sampling. The biomass of the plants, the structure of their leaves and roots, the absorption spectrum of photosynthetic pigments, and the oxidative stress were investigated.

### 4.3. Plant Anatomy—Microscopy

For the anatomical/microscopical investigation, two individuals per treatment were used, at random, and a leaf was detached from each one of them. Small pieces (1 × 1 mm^2^) from an area in the middle of the leaf blade and adjacent to the central vein were excised. 

All these pieces together were fixed in phosphate buffered 3% glutaraldehyde (Merck KGaA, Darmstadt, Germany—pH 6.8) at 0 °C for 2 h and post-fixed in 1% osmium tetroxide (Merck KGaA, Darmstadt, Germany) in phosphate buffer for 2 h at 0 °C. A few pieces were dehydrated in graded acetone series, critical point dried (Autosamdri^®^-815, Tousimis, Rockville, MD, USA), coated with gold, and viewed with a JEOL JSM-6360 scanning electron microscope. The rest of the tissue was dehydrated in a graded ethanol series and embedded in Durcupan ACM (Fluka, Steinheim, Switzerland). Semithin sections obtained from a LKB Ultrotome III (LKB-Produkter AB, Stockholm, Sweden) were placed on glass slides and stained with 0.5 toluidine blue O (in 1% borax solution) as a general stain for light microscopic observations. Sections of epoxy-embedded material were viewed with an OLYMPUS CX-41 Light Microscope (Tokyo, Japan). Original light micrographs were recorded digitally, using a Nikon D5600 camera (Tokyo, Japan) at 24.2 megapixels. The protocols for double fixation, embedding, sectioning, and light microscope observations are cited in detail by Christodoulakis et al. [63]. 

Root samples were also collected from the same two individuals and were embedded using the same protocols for light microscope observation.

### 4.4. Photosynthetic Pigments

The spectral absorbance of photosynthetic pigments was measured in leaf samples taken from four random individuals per treatment. Approximately 50 mg of leaf tissue was sampled from each individual and placed in a tube with 1.5 mL of 80% acetone for 48 h at 4 °C. The supernatant was then transferred to a quartz cuvette, and the absorbance from 400 to 700 nm was recorded in a V-1200 spectrophotometer (VWR International, PA, USA). The protocol used is a modification of the one used by Stefi et al. [28]. The absorbance at 663.2, 646.8, and 470 nm was used to calculate the pigment content of chlorophyll *a*, chlorophyll *b,* and carotenoids. Quantification of pigment content was calculated using the following formulas [64]:

Chlorophyll *a* = 12.25 × A_663.2_ − 2.79 × A_646.8_

Chlorophyll *b* = 21.50 × A_646.8_ − 5.10 × A_663.2_

Chlorophyll *a* + *b* = 7.15 × A_663.2_ + 18.71 × A_646.8_

Total Carotenoids = (1000 × A_470_ − 1.82 × Chl *a* − 85.02 × Chl *b*)/198

The resulting value was normalized per fresh weight. 

### 4.5. Oxidative Stress (H_2_O_2_ and MDA)

Reactive oxygen species (ROS) levels were estimated via lipid peroxidation using the MDA method; 50 mg of frozen plant material deriving from four leaves of four randomly selected individuals per treatment were used. Roots weighing 50 mg were also harvested from four individuals per treatment. The frozen plant material was homogenized and mixed with 1 mL 0.25% thiobarbituric acid (TBA) dissolved in 10% trichloroacetic acid (TCA) solution to extract the MDA. The extract was collected and heated at 85 °C for 30 min. The mixture was centrifuged at 13,000× *g* for 10 min, and the supernatant was transferred to a cuvette for photometry. The absorbance was read both at 532 nm (MDA-TBA peak) and 600 nm (non-specific absorbance). The MDA concentration was estimated using formulas described by Gechev et al. [65]. Total ROS were expressed as moles MDA/g of leaf tissue.

Furthermore, oxidative stress was also measured by the H_2_O_2_ method [66,67]. Moreover, 50 mg of leaf and root tissues also selected randomly as described above were harvested and homogenized in an ice bath with 0.5 mL 0.1% (*w*/*v*) trichloroacetic acid (TCA). The homogenate was centrifuged at 12,000× *g* for 15 min, and then 0.5 mL of the supernatant was added to 0.5 mL of 10 mM potassium phosphate buffer (pH 7.0); 1 mL of 1 M potassium iodide (KI) was also added. The absorbance of the supernatant was read at 390 nm, while the H_2_O_2_ content was calculated via a standard curve.

### 4.6. Isolation of Bacteria

On the 60th day of plants’ cultivation, a total of 10 g of each individual soil sample taken from the rhizosphere was mixed well and divided into two equal parts. From the first part of the samples, 1 g of soil was placed in sterile Falcon tubes, dissolved in 10 mL of Ringer (0.25 strength), and shaken on an orbital shaker at maximum speed (220 rpm) for 1 h. Serial dilution to 10^−5^ was performed, and 0.1 mL of each dilution was spread on three different media plates. The second part of the samples was heat-treated for 1 h in a hot air oven at 60 °C and then managed like the first one. Media used for the cultivation of the isolates were the Arginine Glycerol Salts agar (AGS) [68], the modified Arginine Glycerol Salts agar with 100 mg K_2_Cr_2_O_7_ (AGSCr), to reduce fungal growth, and Nutrient Agar (NA) [69]. After incubation at 30 °C for 72 h, each morphologically distinct bacterial colony was picked up and purified by further cultivation on AGS and NA medium plates. All isolates were maintained as suspensions in 40% (*w*/*v*) glycerol solution at −80 °C. All isolates were deposited in the Athens Collection of Bacteria and Archaea (ATHUBA) and received the corresponding accession number.

### 4.7. In Vitro Antimicrobial Activity 

The antimicrobial activity of the isolated strains was tested by diffusion method against six indicator strains (Table 5). An aliquot of each isolated strain (10 μL) from the glycerol suspension was inoculated into Eppendorf tubes with 500 μL of liquid nutrient AGS or NB, followed by incubation under agitation at 180 rpm at 30 °C for 48 h. Aliquots of 10 μL of each liquid culture were then spot inoculated into Petri dishes with AGS or NA media (4 aliquots per plate). The inoculated plates were incubated at 30 °C for 5 days and then exposed to UV for 20 min to inhibit their further growth. Furthermore, the plates were overlaid by soft medium (Nutrient Agar for the bacteria and Yeast Extract Agar for the fungi indicator strains) containing 0.7% *w*/*v* agar inoculated with 100 μL from a liquid culture of each indicator strain. The plates were then incubated for 48 h at the incubation temperature of the indicator microorganism, and then the diameters of inhibition growth zones were measured. The liquid cultures of the indicator strain were prepared as follows: An aliquot of each indicator strain (10 μL) from a stock glycerol suspension was inoculated into Eppendorf tubes with 500 μL of liquid medium, Nutrient Broth for the bacteria, and Yeast Extract for the fungi strains, followed by incubation under agitation at 180 rpm for 48 h at the proper temperature (Table 5) (modified [70,71]).

### 4.8. Statistical Analysis 

All analyses were performed in triplicate, and the relative standard deviation (RSD %) was estimated for each biological triplicate. The statistical significance of results was evaluated using one-way analysis of variance (ANOVA) of OriginPro v.9.1 (OriginLab Corporation, Northampton, MA, USA). *p* values ≤ 0.05 were characterized as statistically significant. Pigment concentration analysis was performed using the software SPSS Statistics v.22.0 (IBM Corp, Armonk, NY, USA); statistical analysis for multiple comparison testing between groups was performed using the post hoc Tukey test.

## 5. Conclusions

The results of our experiments demonstrate the beneficial role of the soil improvers in biomass production and in supporting the development of beneficial microorganisms that can boost plant defense against pathogens. They are based on a practical example of municipal waste management, according to which the farmer obtains the soil improvers free of charge or at a very low cost while, at the same time, the municipality overcomes the problem of waste accumulation in a green, sustainable way.

Ginagro 5, Ginagro 10, VitaGreen+ 5, and VitaGreen+ 10 greatly enhance the yield at no cost. All improvers significantly increase the chlorophyll content of the leaves while reducing, except for VitaGreen+ 5, their MDA content. Finally, the few bioactive strains isolated from the amended substrates present moderate inhibition of the microbial indicators used. 

Our results strengthen the concept of the conversion of organic waste (zero/non-value) to new, valued, and useful products to boost one of the bioeconomy’s 4F’s (food, feed, fuel, and fiber). This is a big, if not the major, challenge of the circular economy. Soil improvers, amongst many other benefits, are friendly to the environment and improve the structure of the soil. The current investigation seems to groove further the new “way out” and the first, small-scale, positive steps on behalf of a “greener” environment and an “active” bioeconomy. Under this scope, the biotransformation of carbon in soil could also contribute to a partial relief from climatic change CO_2_ “atmospheric symptoms” [42].

## Figures and Tables

**Table 1:** Inhibition (●) of the microbial indicators by the 17 isolated strains with antimicrobial activity.

		Inhibition					
Soil Sample	Strains	*B. subtilis*	*S. aureus*	*P. aeruginosa*	*E. coli*	*C. albicans*	*S. cerevisiae*
Soil	ATHUBA 2566						
	ATHUBA 2567						
	ATHUBA 2568						
	ATHUBA 2569						
	ATHUBA 2570						
Soil + Fertilizer	ATHUBA 2576						
	ATHUBA 2579						
	ATHUBA 2581						
	ATHUBA 2584						
	ATHUBA 2587						
VitaGreen 5%	No bioactive strains						
VitaGreen 10%	ATHUBA 2616						
	ATHUBA 2620						
VitaGreenPLUS 5%	No bioactive strains						
VitaGreenPLUS 10%	ATHUBA 2627						
	ATHUBA 2632						
Ginagro 5%	ATHUBA 2670						
	ATHUBA 2671						
	ATHUBA 2674						
Ginagro 10%	No bioactive strains						

**Table 2:** Main properties of the plain Kalamata soil.

Clay (%)	Sand (%)	Silt (%)	pH	Mg^2+^ (mg/kg)	K^+^ (mg/kg)	Na^+^ (mg/kg)	Ca^2+^ (mg/kg)	P (mg/kg)	N (%)	Total Organic Carbon (%)	Organic Parts (%)	C.E.C. (meq/100 g)
14.2	47.8	38	5.8	197	82	70	1540	7	0.175	2.223	4.446	16.78

**Table 3:** Main properties of the soil improvers.

	Vita Green	Vita Green Plus	Ginagro
Composition	Plant waste	Plant and food waste	Cotton ginner plant waste
Adjustments	Aeration and surface watering	Aeration and surface watering	Aeration and surface watering
Soil Moisture (%)	49.6	49.5	63.49
Conductivity (mS/cm) (1/5)	0.7	2.7	1.8
pH	7.8	7.7	8.62
Relative Density (g/mL)	0.34	0.42	0.32
Ash (%)	32.3	33.3	-
Organic Parts (%)	67.7	63.7	66.8
Total Organic Carbon (%)	39.2	36.9	-
Total Kjeldahl Nitrogen (%)	1.8	3.2	3.6
Humic Acids (%) (maturity index)	5	8.2	8.5
Pathogens (over approved limit)	Negative	Negative	Negative

**Table 4:** Substrates used for each group and their pH at the end of the experiment.

Group No.	Treatment	Abbreviation	pH
1	Soil	S	6.23
2	Soil with a commercial fertilizer (Complesal)	S + F	6.18
3	Soil with 5% *w*/*w* Vita Green compost	VG 5%	7.24
4	Soil with 10% *w*/*w* Vita Green compost	VG 10%	7.45
5	Soil with 5% *w*/*w* Vita Green Plus compost	PLUS 5%	7.04
6	Soil with 10% *w*/*w* Vita Green Plus compost	PLUS 10%	7.23
7	Soil with 5% *w*/*w* Ginagro compost	GIN 5%	7.90
8	Soil with 10% *w*/*w* Ginagro compost	GIN 10%	8.12

**Table 5:** Indicator strains and their cultivation conditions.

Indicator Strains	Media	Incubation Temperature	Accession Number
*Bacillus subtilis*	Nutrient Agar/Broth	30 °C	DSM 10
*Escherichia coli*	Nutrient Agar/Broth	37 °C	NEB dh5a
*Pseudomonas aeruginosa*	Nutrient Agar/Broth	37 °C	DSM 50071
*Candida albicans*	Yeast Extract Agar/Broth	37 °C	DSM 1386
*Staphylococcus aureus*	Nutrient Agar/Broth	37 °C	DSM 346
*Saccharomyces cerevisiae*	Yeast Extract Agar/Broth	30 °C	DSM 1333

## Data Availability

Dataset available on request from the authors.
